# A tetrahelical DNA fold adopted by tandem repeats of alternating GGG and GCG tracts

**DOI:** 10.1038/ncomms6831

**Published:** 2014-12-15

**Authors:** Vojč Kocman, Janez Plavec

**Affiliations:** 1 Slovenian NMR Centre, National Institute of Chemistry, SI-1000 Ljubljana, Slovenia; 2 EN-FIST Centre of Excellence, SI-1000 Ljubljana, Slovenia; 3 Faculty of Chemistry and Chemical Technology, University of Ljubljana, SI-1000 Ljubljana, Slovenia

## Abstract

DNA can form diverse higher-order structures, whose details are greatly dependent on nucleotide sequence. G-rich sequences containing four or more repeats of three guanines are expected to form G-quadruplexes. Here we show that DNA sequences with GGGAGCG repeats found in the regulatory region of the PLEKHG3 gene are capable of forming tetrahelical DNA structures that are distinct from G-quadruplexes. The d(GGGAGCGAGGGAGCG) sequence, VK1, forms a dimer. Two VK1 sequences connected by an adenine residue, VK2, fold into a monomer, which shares identical structural characteristics with the VK1 fold. Their four-stranded architectures are stabilized by four G-C, four G-A and six G-G base pairs. No G-quartets or Hoogsteen-type hydrogen-bonded guanine residues are present and the overall topology is conserved in the presence of Li^+^, Na^+^, K^+^ and NH_4_^+^ ions. Unique structural features include two edgewise loops on each side of the structure stabilized by three G-G base pairs in N1-carbonyl symmetric geometry.

## Abstract (web summary)

The VK1 sequence of the PLEKHG3 gene is expressed in the brain and is linked to the development of autism. Here, the authors present a structural analysis of this gene and found that due to its alternating GGG and GCG repeats, it folds into a tetrahelical structure distinct from G-quadruplexes.

## Graphical abstract

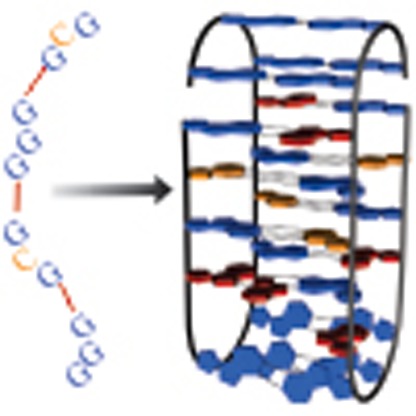

In addition to canonical double-stranded helices, DNA sequences are capable of forming higher-order structures such as G-quadruplexes, i-motifs, hairpins, triplexes and so on, which play an important role in cell biology and are implicated in diseases^1^,^2^. G-rich sequences are known to form G-quadruplexes whose formation has been implicated in essential processes such as initiation of DNA replication, telomere maintenance, regulated recombination in immune evasion and the immune response, control of gene expression and genetic and epigenetic instability^3^,^4^,^5^,^6^,^7^. G-quadruplex structures stabilized by pyridostatin caused transcription- and replication-dependent DNA damage in genomic regions under *in vivo* conditions^8^. Moreover, DNA G-quadruplex structures were quantitatively visualized with the use of engineered, structure-specific antibody probes with high selectivity and low nanomolar affinity^9^,^10^.

High-resolution structures obtained by X-ray crystallography and NMR have shown that G-quadruplexes consist of G-quartets and loop residues of various topologies^11^,^12^,^13^. A large body of structural and biophysical data has demonstrated that three-dimensional (3D) structures of G-quadruplexes are influenced by the nucleotide sequence, cation concentration and external factors such as crowding conditions^14^,^15^,^16^,^17^. A general consensus G-quadruplex motif consists of at least four repeats of three guanine residues separated by loops of various lengths. Over 375,000 sequences with potential to form G-quadruplex structures with loops of 1–7 nt and over 700,000 sequences allowing loops up to 12 nt represent the so-called G4 genome^7^,^18^,^19^,^20^,^21^. Potential G-quadruplex-forming regions in the human genome are not distributed randomly, but are located predominantly in key regulatory regions, such as promoters, gene bodies and origins of replication.

Here we expand the view that four-stranded architectures are formed mostly by sequences of consecutive G-tracts. The d(GGGAGCGAGGGAGCG) sequence, VK1, is found in the regulatory region of the PLEKHG3 gene in the 14th human chromosome. This gene is expressed in many regions of the brain and represents a potential candidate contributing to the risk of autism^22^,^23^. The appearance of GGG and GCG repetitive units in VK1 generates an interesting symmetry with respect to the A residue in the centre of the sequence. From a structural point of view, this tandem repeat sequence is interesting, because it does not adhere to the established G-quadruplex motif. The expected four-stranded structure could consist of a GCGC-quartet sandwiched between a couple of G-quartets^24^,^25^,^26^. However, preliminary ^1^H NMR spectra of the 15 nt construct, VK1, did not suggest formation of G-quartets in a folded structure. The other interesting feature of VK1 is its folding in the presence of Li^+^ ions. It is well known that the folding of a G-rich oligonucleotide into a well-defined G-quadruplex structure requires the presence of K^+^, Na^+^ or other mono- or multivalent cations. These highly unexpected initial observations motivated us to perform a detailed structural study of VK1 as well as its longer 31 nt analogue VK2, formally VK1-A16-VK1, and contribute to understanding of their potential relevance in the occurrence of autism.

## Results

### Identification of a unique fold

**Figure 1:**
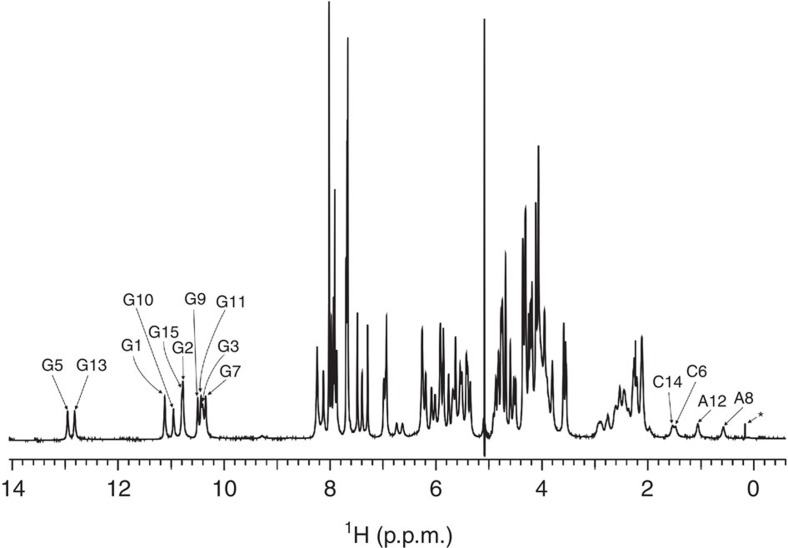
^1^H NMR spectrum of VK1. The assignment of imino protons of guanine residues and C14 H2′, C6 H2″, A12 H2″ and A8 H2″ protons is indicated. A signal of unknown impurity is labelled with *. The spectrum was recorded at 2.8 mM oligonucleotide concentration per strand, 100 mM LiCl, pH 6 and 0 °C on an 800 MHz spectrometer.

We have recorded NMR spectra of the 15 nt sequence d(GGGAGCGAGGGAGCG), VK1, in the absence of K^+^ as well as Na^+^ ions and at low and high Li^+^ ion concentrations. Perusal of a downfield region of ^1^H NMR spectrum of VK1 in 100 mM LiCl shows 10 sharp and well-resolved imino proton resonances (Fig. 1).

Two of the imino proton resonances with chemical shifts of *δ*12.82 and 12.95 p.p.m. are characteristic for imino protons of G-C base pairs associated in Watson–Crick geometry. The other eight resonances appear between *δ*10.3 and 11.2 p.p.m. and are indicative of imino protons involved in uncanonical hydrogen bonds between guanine residues. These signals could be assigned to Hoogsteen-type hydrogen bonds that are normally associated with the formation of G-quartets. However, their presence in the spectrum of VK1 shown in Fig. 1 is surprising, since it is known that G-quartet formation is not possible in the presence of Li^+^ ions alone. The imino proton resonances of VK1 were unambiguously assigned with the help of ^15^N-edited HSQC (heteronuclear single-quantum correlation) spectra acquired on nine partially (6%) residue-specific ^15^N-labelled oligonucleotides (Supplementary Fig. 1)^27^,^28^. All 10 guanine imino protons are hydrogen bonded and each of the imino proton resonances is assigned to a single guanine residue.

**Figure 2:**
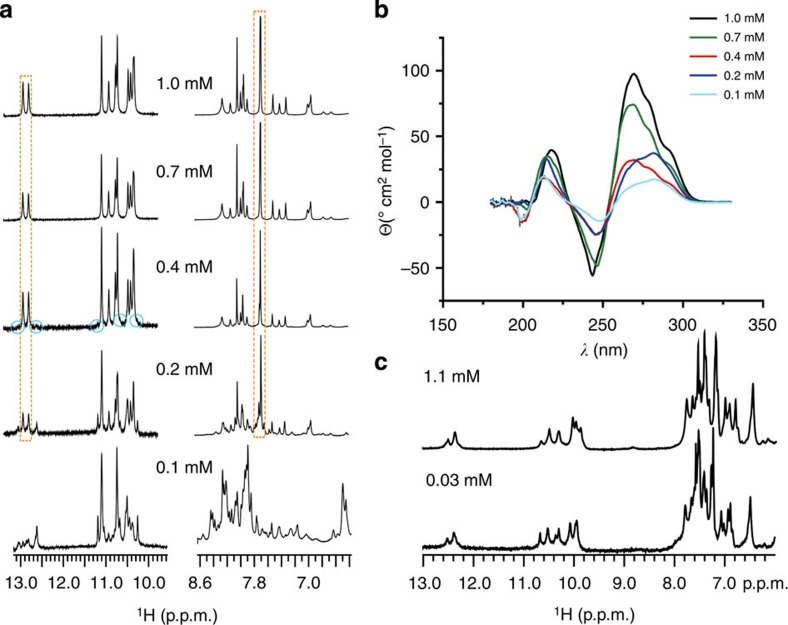
Concentration-dependent ^1^H NMR and CD spectra. (**a**) ^1^H NMR spectra of VK1 at 0 °C, pH 6 and different oligonucleotide concentrations indicated next to individual spectra. The most characteristic signals of the VK1 fold are marked with a dashed square. The blue circles mark small signals that appear at 0.4 mM concentration and indicate a different fold. (**b**) CD spectra of VK1 at different concentrations per strand. The spectra were recorded in a 0.25-cm path-length cell at 0 °C, 100 mM LiCl and pH 6. (**c**) ^1^H NMR spectra of VK2 at 0 °C, pH 6 and 0.03 and 1.1 mM oligonucleotide concentrations.

^1^H NMR and circular dichroism (CD) spectra of VK1 are strongly dependent on oligonucleotide concentration (Fig. 2a,b). The NMR spectra show that a single (VK1) fold is present only at concentrations above 0.4 mM per strand. At 0.4 mM oligonucleotide concentration, NMR signals of low intensity that indicate the presence of different folds start to appear.

NMR signals indicative of different folds get more numerous and intense on lowering the oligonucleotide concentration to 0.2 mM. In parallel to NMR, CD spectra recorded at 0.1 and 0.2 mM concentrations are significantly different than at higher concentrations (Fig. 2b). At these lower concentrations, we identified NMR resonances that corresponded to the folded form as well signals characteristic for the single-stranded form. These signals were used to perform a study of the concentration dependence of the folded form relative to the single-stranded form^27^. The slope of a graph in Supplementary Fig. 2 yielded a strand stoichiometry for the folded form of 2.0±0.5. In addition, the translational diffusion coefficient of VK1 in 90% H_2_O, 10% D_2_O at 0 °C and 2.8 mM oligonucleotide concentration per strand was 0.40 × 10^−10^ m^2^ s^−1^. The corresponding hydrodynamic diameter of 44 Å approximated using the spherical model and assuming thickness of the hydration layer (2.8 Å) is consistent with a structure comprised of around nine stacked planes. The translational diffusion coefficient, stoichiometry determination and concentration-dependent ultraviolet melting temperatures agree with a dimeric structure. Since we have observed only a single set of signals in the ^1^H NMR spectra, we can conclude that VK1 folds into a dimeric structure that is highly symmetric.

**Figure 3:**
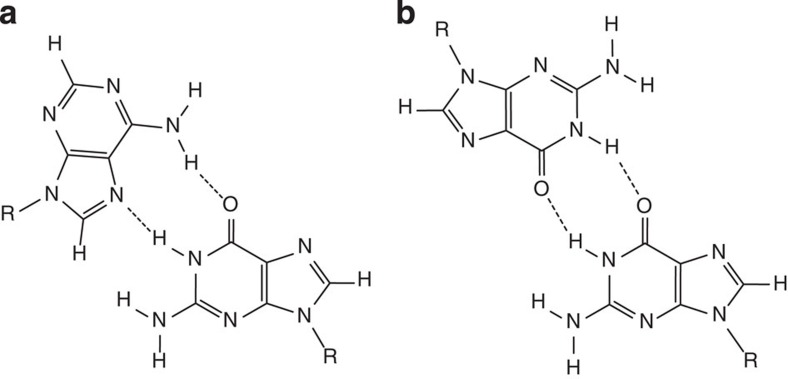
Noncanonical G-A and G-G base pairs. (**a**) Schematic presentation of G-A base pair in N7-N1 amino carbonyl geometry. (**b**) G-G base pair in N1-carbonyl symmetric geometry.

Further analysis of NMR data has shown that the structure consists of four G-C base pairs in Watson–Crick geometry, four noncanonical G-A and six G-G base pairs (Fig. 3). The correlation signals in the NOESY (nuclear Overhauser effect spectroscopy) spectra reveal that the G-A base pairs adopt a N7-N1 amino carbonyl geometry. The G-G base pairs adopt a N1-carbonyl symmetric geometry, which is the only known arrangement of such base pairs that is C2 symmetric and allows for imino protons of both guanines to be hydrogen bonded.

**Figure 4:**
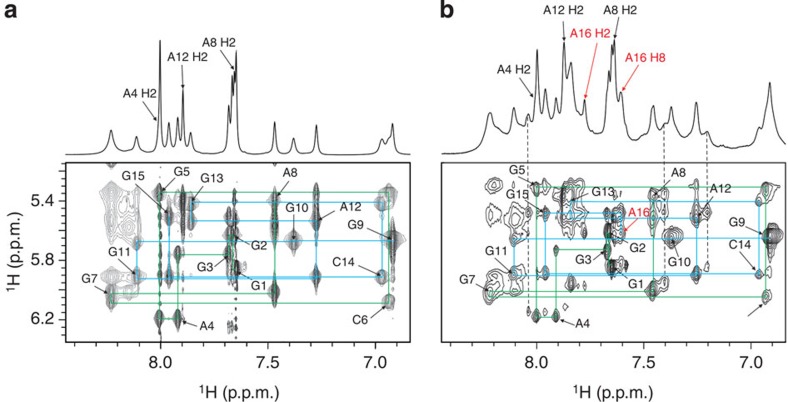
Sequential assignment of NOESY spectra of VK1 and VK2. Aromatic regions of ^1^H NMR spectra (upper panels) and anomeric–aromatic regions of NOESY spectra of VK1 (**a**) and VK2 (**b**) constructs at pH 6.0, 0 °C and mixing time of 80 ms. The spectra of VK1 and VK2 were recorded at 2.8 and 1.0 mM oligonucleotide concentration per strand, respectively. Signals of H2 adenine protons are labelled in black. The sequential walks from G1 to A8 and from G9 to G15 are depicted in green and blue, respectively. (**b**) H2 and H8 resonances of the linker A16 residue are marked in red. The vertical dashed lines indicate resonance frequencies of H8 protons of G15, A8 and A12, which point to distortions in symmetry of VK2.

Perusal of the upfield region of the ^1^H NMR spectrum shows four signals dispersed over a spectral range between *δ*0.5 and 1.6 p.p.m. with surprisingly low chemical shifts for C14 H2′, C6 H2″, A12 H2″ and A8 H2″ protons (Fig. 1). The unusual spectral features made proton assignment challenging. H8 proton resonances of G1, G2, G3, G9 and G10 were assigned through two-bond couplings with N7 atoms with the help of 1D ^15^N-edited spectra recorded on partially (6%) residue-specific ^15^N-labelled oligonucleotides (Supplementary Fig. 3). Assignment of H8 proton resonances was confirmed unambiguously with the use of completely (100%) D8 residue-specific labelled oligonucleotides. Of particular importance was the identification of the aromatic protons that were absent in NOESY spectra of D8-labelled VK1 at positions G5 and G7 (Supplementary Fig. 4). Utilizing the above structurally independent assignments, we were able to unambiguously assign all aromatic ^1^H resonances of VK1 in a NOESY sequential walk. Intensity of cross-peaks in the aromatic–anomeric region of NOESY spectra of VK1 and sequential connectivities observed between H1′(*n*) and H8/H6(*n*+1) protons were in complete accordance with anti-orientations across glycosidic bonds of all nucleotides (Fig. 4a). The assignment of imino and aromatic protons as well as the vast majority of ^1^H resonances was highly complete, including some of the H4′, H5′ and H5″ sugar protons.

### The NOE contacts reveal an unprecedented symmetric topology

**Figure 5:**
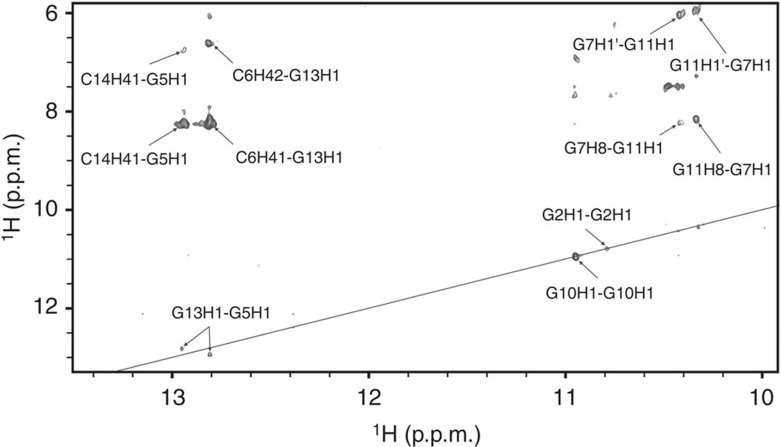
The imino–imino region of ^15^N-edited and ^15^N/^13^C-filtered NOESY spectrum. Data were acquired on a sample that contains an equimolar mixture of unlabelled and uniformly ^13^C, ^15^N-GC-labelled VK1 oligonucleotides. The spectrum was recorded at 0.9 mM concentration per strand, 0 °C, pH 6 and mixing time of 100 ms. Assignment of individual cross-peaks is indicated. The position of a diagonal in a corresponding standard ^1^H–^1^H NOESY spectrum is shown as a straight line.

G5-C14 and C6-G13 base pairs were identified by observing characteristic NOE cross-peaks between both cytosine amino protons and guanine H1 proton in the Watson–Crick chemical shift range. NOE cross-peaks between adenine H8 and guanine H1 protons confirmed the formation of A4-G15 and G7-A12 base pairs in N7-N1 amino carbonyl geometry. All G, C and A residues from one molecule, except for the two A8, are involved in hydrogen bonding with the residues from the other molecule. As the VK1 fold is highly symmetric, it became crucial to distinguish between inter- and intramolecular NOE contacts. VK1 sample with uniformly ^13^C, ^15^N-labelled guanine and cytosine residues was synthesized and used to prepare an equimolar mixture of unlabelled and uniformly ^13^C, ^15^N-GC-labelled oligonucleotides. This sample was used to record a ^15^N-edited, ^15^N,^13^C-filtered NOESY spectrum that allowed observation of only intermolecular NOE contacts between protons bound to ^15^N nuclei and protons bound to ^14^N and ^12^C nuclei (Fig. 5)^27^,^29^.

**Figure 6:**
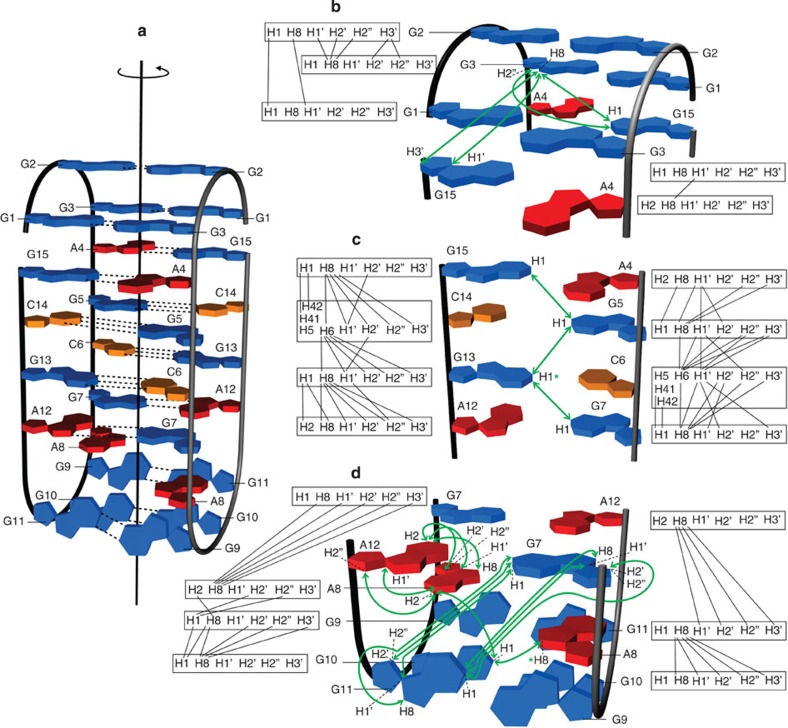
The topology of the tetrahelical structure adopted by VK1. The guanine bases are coloured in blue, adenine in red and cytosine in orange. Hydrogen bonds are shown as dotted lines. (**a**) The entire topology with a shown C_2_ axis of symmetry. (**b**) NOE connectivities of G1-G3, G2-G2, G3-G1 and A4-G15 base pairs. (**c**) NOE connectivities between A4-G15, G5-C14, C6-G13 and G7-A12 base pairs. (**d**) NOE connectivities of G9-G11, G10-G10, G11-G9, G7-A12 base pairs and two A8 residues. The green arrows connect protons with a NOE cross-peak between non-sequential residues. The green asterisks highlight the G13 H1 and A8 H8 protons that exhibit intermolecular NOE cross-peaks. The black lines represent NOE cross-peaks between sequential residues. Intra-residue NOE connectivities and NOE cross-peaks between G-C and A-G base pairs are omitted for clarity.

The ^15^N-edited, ^15^N,^13^C-filtered NOESY spectrum conclusively shows that the G5-C14 and C6-G13 Watson–Crick base pairs are formed between two molecules of VK1 (Fig. 5; Supplementary Fig. 5). The G13H1-G5H1 NOE contact is consistent with the stacking of the sequential G-C base pairs. The G2H1-G2H1 and G10H1-G10H1 NOE contacts are the only contacts that are positioned on the supposed diagonal. Such contacts are only possible in the presence of G2-G2 and G10-G10 base pairs in N1-carbonyl symmetric geometry, which must exist between the two strands. The G7H1-G11H1′, G7H1-G11H8, G7H1′-G11H1 and G7H8-G11H1 NOE contacts suggest that G7 is spatially close to G11 from the other strand (Fig. 6a,d).

The intermolecular NOE contacts gathered from the ^15^N-edited, ^15^N,^13^C-filtered NOESY spectrum together with NOE interactions from the ^1^H–^1^H NOESY spectrum enabled us to build a symmetric VK1 topology consistent with all inter- and intrastrand distances. In the VK1 topology, each of the four strands is surrounded by two neighbouring strands that are oriented in an antiparallel manner. The orientation of the four strands in the bimolecular topology is in full agreement with a C_2_ axis of symmetry running through the core of the structure. G1-G3, G2-G2 and G3-G1 interstrand base pairs stabilize two loops each containing the 5′ ends of two VK1 molecules (Fig. 6b). The two loops in edgewise orientation place the two G3-G1 base pairs close to two A4-G15 base pairs. G3 residues exhibit NOE contacts with both G15 residues. Two sets of intermolecular A4-G15, G5-C14, C6-G13 and G7-A12 base pairs comprise the symmetric core of the structure, which is well defined by ample NOE cross-peaks (Fig. 6c). The imino–imino walk along H1 protons of G residues involved in G-A and G-C base pairs confirms the intermolecular nature of G15·A4, G5-C14, G13-C6 and G7-A12 base pairs and shows that the base pairs are involved in efficient stacking interactions.

Two loops formed by the central residues of VK1 adopt edgewise orientations and are stabilized by G9-G11, G10-G10 and G11-G9 intermolecular base pairs (Fig. 6d). From the NOE contacts between H1 of G7 from one molecule and H1′, H2′, H2″ and H8 protons of G11 from the other molecule, and vice versa, we can conclude that G7 residue is very close and possibly even stacked on G11 from the other molecule.

The intramolecular NOE contacts between G7 and G11 are not observed, which is in agreement with the position of A8 between the two residues of the same molecule. A8 is spatially close to A12 from the same molecule, which is supported by intramolecular NOE cross-peaks between A8 H2 and A12 H1′ and H2″ as well as between A12 H2 and A8 H1″, H2″, H2″ and H8 protons. G10 H1 exhibits an intramolecular NOE cross-peak with A8 H2 and intermolecular contact with A8 H8. G9-G11, G10-G10 and G11-G9 base pairs form a pocket for the two A8 residues, which are additionally stabilized by a couple of G7-A12 base pairs. The position of the A8 residue is also determined by the intermolecular NOE cross-peak observed between G13 H1 and A8 H8 protons. A C_2_ axis of symmetry runs through the centre of the G2-G2 and G10-G10 base pairs at the opposite ends of the structural model (Fig. 6a).

Oligonucleotides with A4 to T4, A8 to T8 and A12 to T12 mutations as well as the mutant of VK1 with all adenine residues changed to thymine residues exhibit significantly different ^1^H NMR spectra with respect to VK1 (Supplementary Fig. 6).

**Figure 7:**
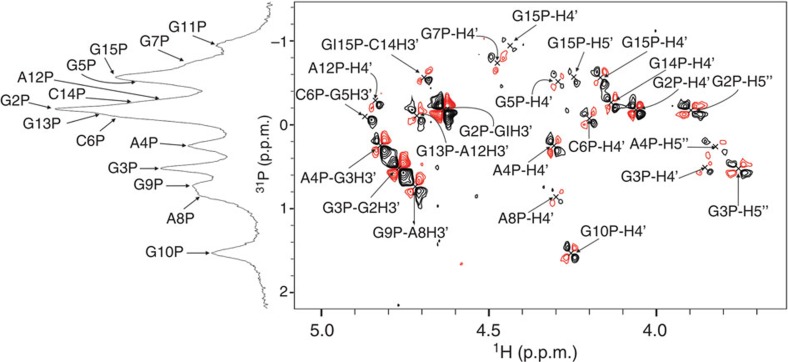
1D ^31^P NMR spectrum shown along a 2D ^1^H–^31^P COSY spectrum. Assignment of individual cross-peaks is indicated. Both spectra were recorded at 1.0 mM oligonucleotide concentration per strand, pH 6 and 0 °C.

Unusual structural features of a sugar–phosphate backbone are well correlated with (de)shielding of ^31^P resonances that are sensitive to differences in O–P–O bond angels and backbone torsions. The 14 signals in 1D ^31^P spectrum are located in a chemical shift region between *δ* −1.2 and 1.8 p.p.m. (Fig. 7). The phosphorus atoms were assigned through their long-range scalar couplings with H3′(*n*) as well as H4′(*n*+1) and H5′/H5″(*n*+1) protons in a ^1^H–^31^P COSY spectrum. From the characteristic cross-peak splitting observed in the ^1^H–^31^P COSY spectrum, it was deduced that the backbone torsion angles *ε* (C4′-C3′-O3′-P) of G1, G2 and G3 and *β* (P-O5′-C5′-C4′) of G2, G3 and A4 residues are in gauche(-) conformation (Fig. 7).

### Guanines might not be G-quartet's best friends

**Figure 8:**
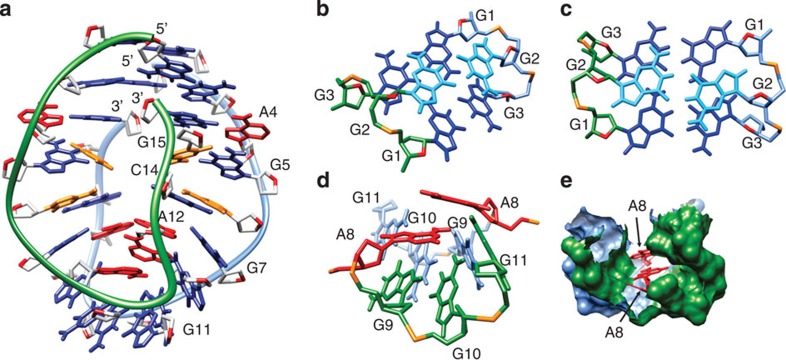
A view of the lowest energy structure of the VK1 construct. The guanine bases are coloured blue; adenine, red; cytosine, orange; sugar, light gray or the same color as the strand; O4′ atoms, red; and phosphorus atoms, orange. The two strands are coloured green and light blue. In **b**,**c**, the two G2 residues are coloured cyan. (**a**) The representation of the overall structure. (**b**) Side view of G1-G3, G2-G2 and G3-G1 base pairs. (**c**) Top view of G1-G3, G2-G2 and G3-G1 base pairs. (**d**) Orientation of two A8 residues and G9-G11, G10-G10 and G11-G9 base pairs. (**e**) Two A8 residues are accommodated inside the hydrophobic pocket. The surfaces of the two strands are coloured with green and blue.

Three hundred and twenty-four NOE-derived distance restraints together with 70 torsion angles and 32 hydrogen-bond restraints were used to calculate the high-resolution structure of VK1 (Fig. 8). The calculations carried out in the presence of explicit water molecules resulted in a well-converged family of structures and minor deviations from restraints obtained from NMR data. A family of 10 final structures of VK1 exhibits a pairwise heavy atom r.m.s.d. (root mean squared deviation) of 1.1 Å (Table 1; Supplementary Fig. 7).

All residues exhibit anti-glycosidic torsion angle values. Four G-C base pairs are located in the centre of the structure. There are no GCGC-quartets, which could in principle occur through association of neighbouring G-C base pairs via minor or major grooves^24^,^25^. The fold of VK1 could theoretically facilitate hydrogen bonding between G and C base pairs only through their major groove sides. The large chemical shift differences (Δ*δ* ~1.4 p.p.m.) in amino proton resonances of C6 and C14 suggest that only one of them is hydrogen bonded, which precludes a direct major groove GCGC-quartet alignment. The slipped major groove GCGC-quartet alignment has been stabilized by K^+^ ion interactions in d(G_3_CT_4_G_3_C), a sequence originating from adeno-associated viral DNA^30^. In the structure of VK1, the two G5-C14 base pairs are separated to an extent that prevents them from forming hydrogen bonds with one another. The same applies for the two C6-G13 base pairs. The distance between respective O6 and N4 atoms exceeds 6 Å in both cases and thus creates a cavity in the core of the structure. In the family of final structures, a buckle experienced by the two G5-C14 base pairs is between 20° and 40°, whereas the buckle is close to 0° for the two C6-G13 base pairs. The neighbouring G5-C14 and C6-G13 base pairs experience a roll of about 40°. The four central G-C base pairs are sandwiched by the four G-A base pairs. The rise between the base pairs in the core of the structure is ~3.3 Å. In the family of final structures, the buckle for the two A4-G15 and G7-A12 base pairs is close to 6° and between 20° and 30°, respectively. The two A4-G15 base pairs experience a high propeller twist of up to 30°. The increased buckle and propeller twist of A4-G15, G5-C14, C6-G13 and G7-A12 base pairs together with backbone curvature result in a cavity in the core of the structure (Fig. 8).

An interesting structural feature of VK1 is the mutual arrangement of sequential residues that define stacking patterns. Stacking of residues from A4 to G7 in the same molecule continues on G11 from the other molecule. The other stacking pattern starts with A12 and continues to G15 in the same molecule (see, for example, Figs 6 and 8). The two stacking patterns are separated by a G10-G10 base pair.

The G1–G3 tracts adopt an antiparallel fold-back topology consisting of three G-G base pairs in N1-carbonyl symmetric geometry. Loop-like features of this segment of the structure of VK1 are correlated with gauche(-) conformation of *ε* and *β* torsion angles within G1-G2-G3-G4 segments. The two G1-G3 base pairs delineate a plane. The G2-G2 base pair is positioned on top of this plane (Fig. 8b,c). Such spatial distribution makes the G1, G2 and G3 imino protons exposed to the surrounding solvent, which is evident through observed exchange cross-peaks with bulk water in a ROESY (rotational nuclear Overhauser effect spectroscopy) spectrum (Supplementary Fig. 8). H8 proton signals of G1, G2 and G3 are positioned in the middle of the H8 chemical shift region and exhibit very small chemical shift dispersion, which correlates very well with the weak stacking experienced by the G1-G3, G2-G2 and G3-G1 base pairs (Figs 4a and 8c).

On the opposite side of the structure, the G9-G11, G10-G10 and G11-G9 base pairs are also arranged in a fold-back topology around two A8 residues. The G9, G10 and G11 residues experience large fluctuations in backbone geometry (Fig. 8d), which is reflected in a clear chemical shift dispersion of G9P, G10P and G11P phosphorus signals (Fig. 7). In the family of final structures, a buckle as well as a propeller twist of about 10° are experienced by the two G9-G11 base pairs. The two A8 residues are tightly packed with G9-G11, G10-G10, G11-G9 and two G7-A12 base pairs (Fig. 8e). This stabilization by hydrophobic interactions protects exchangeable protons of A8 from exchange with water molecules. In the family of 10 final structures, the distance between A8 H8 and the G9 sugar protons is too long for NOE cross-peaks to be observable. This is in accordance with the interrupted sequential walk between residues A8 and G9 in the NOESY spectrum (Fig. 4a).

### Upfield chemical shifts of H2′ and H2″ protons

The two A8 residues are surrounded by tightly packed residues from all sides, which determine chemical shifts of their protons through ring currents of aromatic moieties. The extensive shielding of A8 H2″ is reflected in its upfield chemical shift (*δ*0.58 p.p.m.; Fig. 1). Similarly, A12 is surrounded by two A8 residues as well as G9-G11 and C6-G13 base pairs, which leads to strong shielding of its H2″ (*δ*1.17 p.p.m.). Likeness of chemical shifts of C6 H2″ (*δ*1.48 p.p.m.) and C14 H2′ protons (*δ*1.53 p.p.m.) and strong shielding with respect to the standard chemical shift range can be explained by effective interactions and stacking of base pairs in the core of the tetrahelical structure. Assessment of distances in the structure of VK1 shows that the C6-G13 base pair is close to the two A8 residues and G7-A12 base pair, which affects shielding of C6 H2″. The G5-C14 base pair is further away, which is in accordance with a slightly higher chemical shift value of the C14 H2′ proton.

### Cation insensitivity of the VK1 fold

**Figure 9:**
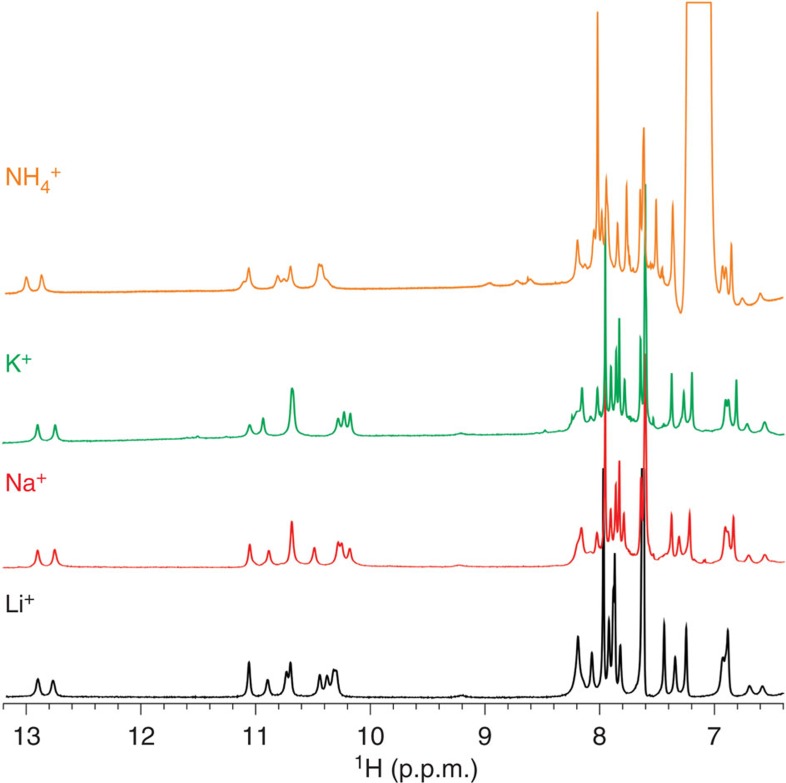
^1^H NMR spectra of VK1 in the presence of different cations. The spectra were recorded at 2.0 mM oligonucleotide concentration per strand, pH 6, 0 °C and 100 mM cation concentrations on an 800 MHz spectrometer.

According to minor variations in ^1^H NMR spectra, the same fold is observed in the presence of Li^+^, Na^+^, K^+^ and NH_4_^+^ ions (Fig. 9 and Supplementary Fig. 9 for CD spectra). Examination of potential cation-binding sites in VK1 shows no structural motifs that would require cations for their stabilization. Such behaviour is in complete contrast to G-quartet-based structures that are not formed in the absence of cations such as K^+^ and Na^+^. The current study expands our understanding of the role of cations in folding of G-rich DNA sequences. G-repeats involved in G-G N1-carbonyl symmetric base pairs should be taken into account when contemplating possible G-rich DNA folds. It should also be considered that such non-B DNA structures might be more frequent within genomes than generally excepted.

### Double-repeat construct folds into a very similar structure

**Figure 10:**
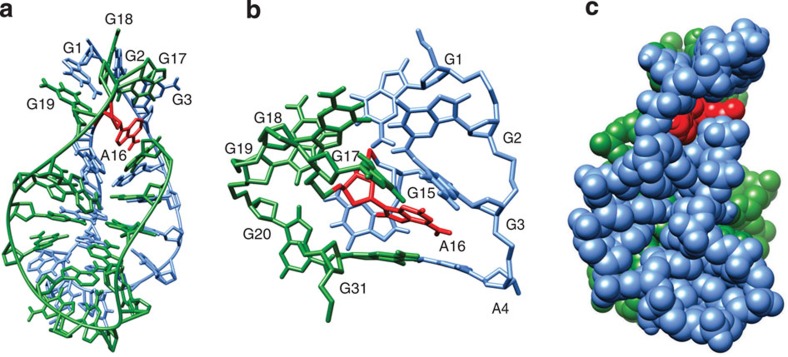
A view of the VK2 structure. Residues G1 to G15 and G17 to G31 are coloured in light blue and green, respectively. The A16 residue is coloured in red. (**a**) A representation of the overall structure of VK2. (**b**) Enlarged view of the A16 residue and the two loops stabilized by G-G base pairs. (**c**) The surface representation of the structure.

Knowing that VK1 folds into a dimeric structure with 5′ and 3′ ends being predisposed for connecting led us to design a VK2 construct in which two 15 nt sequences of VK1 are connected by a single A residue (that is, sequence of 31 nt formally consists of VK1-A16-VK1). Simple 1D NMR and NOESY spectra of VK2 showed almost identical fingerprint signals as observed in VK1 spectra in all spectral regions (Fig. 4; Supplementary Fig. 10). The only differences were due to the additional signals assigned to the A16 residue in VK2 and doubling of parts of the sequential walk as a result of distortions in symmetry (see, for example, Figs 4a and 3b). Comparison of NMR spectra and their detailed analysis showed that VK2 adopts a structure that is very similar to VK1, albeit unimolecular. In brief, the A16 residue in VK2 enables an intramolecular fold by formally connecting the 5′ and 3′ ends of individual molecules of VK1 (Fig. 10).

As comparative analysis of VK1 and VK2 NMR spectra showed great similarities, we could use the calculated structure of the VK1 with the added A16 residue as a starting structure. NOE-derived distance restraints were gathered from VK2 NOESY spectra. Simulated annealing protocol in addition included torsion angle and hydrogen-bond restraints to calculate a model of a VK2 construct (Fig. 10). The final structure of VK2 is very similar to the structure of VK1 with the exception of A16 linkage. The A16 residue in VK2 interferes with the arrangement of G1-G19(G3), G2-G18(G2) and G3-G17(G1) base pairs, which represents the most noticeable difference between VK1 and VK2 structures. In VK1, the G1-G3, G2-G2 and G3-G1 base pairs are planar (Fig. 8a). In VK2, the G2-G18(G2) base pair experiences an increased buckle and the G3-G17(G1) base pair is staggered (Fig. 10b). Examination of the overall fold and solvent-exposed surfaces shows that VK2 structure is very well packed (Fig. 10c). In comparison with VK1, there is no opening in the centre of the structure in the VK2 model.

In contrast to VK1, NMR spectra of VK2 exhibit no concentration dependence (Fig. 2c). ^1^H NMR spectra at 1.1 and 0.03 mM concentrations per strand exhibit only signal characteristics for the VK2 fold. Such behaviour is expected since VK2 adopts a monomeric structure (Fig. 2c). In complete agreement, ultraviolet melting temperature of VK2 is concentration independent, whereas VK1 exhibits concentration-dependent melting temperatures.

### CD signatures of G-G base pairs in VK1 and VK2

At the very outset of the study, the CD spectra of VK1 and VK2 ruled out the formation of parallel and antiparallel G-quadruplexes. In the case of a parallel G-quadruplex, a maximum at 260 nm would dominate. For an antiparallel G-quadruplex, a dominating minimum at 260 nm and two maxima at 240 and 295 nm would be characteristic. In contradistinction, both VK1 and VK2 CD spectra have clear minima at around 245 nm and maxima at around 270 nm, which are signatures of a right-handed stacked helix^31^ (Supplementary Fig. 11). CD spectrum of VK1 has two shoulders at 280 and 290 nm. One shoulder was assigned tentatively to the two loops on one side of the structure stabilized by G1**-**G3, G2**-**G2 and G3**-**G1 base pairs and the other shoulder to the two loops on the other side of the structure stabilized by G9**-**G11, G10**-**G10 and G11**-**G9 base pairs, which are arranged around two A8 residues. When we compare the VK1 and VK2 folds, we can see that the two loops stabilized by G**-**G base pairs, which are arranged around two adenine A8(A24) residues, are structurally very similar. These two loops stabilized by G9**-**G11, G10**-**G10 and G11**-**G9 base pairs in VK1 and by G9**-**G27, G10**-**G26 and G11**-**G25 base pairs in VK2 probably correspond to maximum at around 280 nm. The two loops on the opposite side of the structures are clearly different, since in VK1 the 5′ ends are free, whereas in VK2 the 5′ and 3′ ends of formally individual molecules of VK1 are connected by A16. In VK1, these loop regions are stabilized by G1**-**G3, G2**-**G2 and G3**-**G1 base pairs and possibly correspond to a shoulder around 290 nm. In VK2, the loops connected by the A16 residue are stabilized by G1**-**G19, G2**-**G18 and G3**-**G17 base pairs and likely correspond to a distinct maximum at 295 nm.

### Temperature-dependent CD and NMR spectra of VK1 and VK2

**Figure 11:**
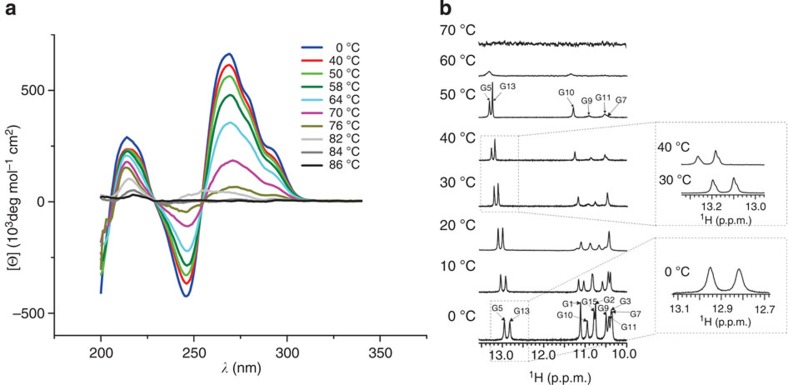
Temperature-dependent CD and NMR spectra. (**a**) CD spectra of VK1 construct were recorded at 0.8 mM concentration per strand in a 0.01-cm path-length cell at 100 mM LiCl, pH 6 and different temperatures indicated by different colours. (**b**) ^1^H NMR spectra of VK1 at 2.8 mM concentration, pH 6 and different temperatures indicated next to individual spectrum. (**c**) The enlarged region characteristic for imino protons of G-C base pairs in Watson–Crick geometry in spectra recorded at 0, 30 and 40 °C.

From the characteristic features of the CD melting experiment, we can observe that the VK1 construct retains the fold even at 76 °C, but is altered at 82 °C (Fig. 11a). In contrast, on increasing the temperature of NMR sample of VK1, local unfolding and disappearance of imino proton resonances of G1, G2 and G3 residues start at 30 °C (Fig. 11b). Imino proton resonances of G5, G7, G9, G10, G11 and G13 residues were still observed at 50 °C. At 60 °C, two broad signals were detected. No imino proton resonances were observed at 70 °C. Comparison of the imino proton resonances showed that the Watson–Crick imino protons exhibit the highest temperature stability. Interestingly, four signals can be observed in the Watson–Crick chemical shift range at 30 and 40 °C (Fig. 11c). These signals point to local structural changes that distort the symmetrical arrangement of the G-C base pairs. In addition to shoulders of G5 and G13 imino protons, there is also a clear shoulder observed for the C6 H6 proton resonance at 40 and 45 °C, while splitting of other individual resonance lines is within the signal linewidth or is obscured due to signal overlap. Noteworthy, lineshapes of signals that do not exhibit shoulders at elevated temperatures display the expected Lorentzian type of signal.

### Implications for G-rich DNA sequences

In contrast to the expected G-quartet-based structure where two quartets were supposed to sandwich a GCGC-quartet, we found a novel four-stranded structure of G-rich DNA that extends the current repertoire of known DNA structures. The unique fold is stabilized though a fine balance of hydrogen bonding and aromatic stacking interactions. Earlier quantum mechanical calculations have suggested that G-G N1-carbonyl symmetric base pairs are of comparable stability to G-C Watson–Crick base pairs if only hydrogen bonds are considered^32^,^33^. Interestingly, a G-G base pair in Hoogsteen geometry is unstable when not part of a G-quartet. Six G-G N1-carbonyl symmetric base pairs are found in the structure of VK1. High stability of these base pairs enables fold-back topologies of G1-G3 and G9-G11 loop regions, which are surprising elements in a G-rich DNA structure where a GGG tract is commonly expected to be involved in G-quartets. Moreover, G9-G11 residues are tightly packed around two A8 residues and additionally stabilized by hydrophobic interactions.

Single d(GGGAGCGAGGGAGCG) sequence, VK1, repeats can be found in 1st, 2nd, 7th, 12th and 21st human chromosomes^34^. In addition, the regulatory region of the PLEKHG3 gene in the 14th human chromosome of the human genome includes three consecutive VK1 repeats. Noteworthy, the PLEKHG3 gene is a potential candidate contributing to the risk of autism. Three VK1 repeats are present in the noncoding strand of the regulatory region, which includes the 5′ UTR (untranslated region) of the PLEKHG3 gene. A single C/A point mutation, insertion of CGAGGGAG and truncations of GAGGGAGC or AGGCGAGG within the three repeats were observed^34^. The mutations can shift the position of the tetrahelical structure that could influence the length of the transcribed 5′ UTR of the PLEKHG3 gene and thus affect the translation of the gene (Supplementary Fig. 12).

Extensive bioinformatics search of genomes of several species showed that the 14th chimpanzee chromosome in the regulatory region of the chimpanzee PLEKGH gene encompasses multiple overlapping VK1 and five consecutive VK1 sequences. In the rest of the chimpanzee chromosomes, mostly single repeats and two overlapping repeats of the VK1 sequence in coding and noncoding strands can be found. In the mouse genome, the VK1 sequence is more prevalent than in the chimpanzee genome and there are more chromosomes with two or three overlapping repeats. In addition, several consecutive VK1 sequences are found in the mouse genome. It is important to highlight mice chromosomes 6 with 10 overlapping VK1 sequences, and 8 with 12 overlapping and 8 consecutive VK1 sequences. In the rat genome, the VK1 sequence is less prevalent than in the mouse genome. In addition to chromosomes with a single VK1 sequence repeat, we can also find seven, four and three overlapping VK1 repeats in rat chromosomes 8, 18 and 5, respectively. Four consecutive VK1 sequences are found in rat chromosome 7 and two consecutive VK1 sequences in rat chromosomes 5 and 18. In the rice genome, we can find one repeat of the VK1 sequence in the coding strands of the 3rd, 4th, 6th, 7th, 10th and 11th as well as in the noncoding strand of the 1st chromosome. Most of the inspected microbial genomes exhibit only one VK1 sequence. However, in the genome of *Blastococcus saxobsidens*, six VK1 sequences spaced apart by hundreds of nucleotides can be found^34^.

In summary, we report on a new, completely unexpected 3D structure of DNA oligomer with the sequence d(GGGAGCGAGGGAGCG), VK1, folded into a well-defined symmetric dimer. The tetrahelical DNA structure is very distinct from an expected G-quadruplex structure. No G-quartets or Hoogsteen-type hydrogen-bonded guanine residues are present and the overall topology is conserved in the presence of Li^+^, Na^+^, K^+^ and NH_4_^+^ ions. Its four-stranded architecture is stabilized by four G-C base pairs in Watson–Crick geometry, four G-A base pairs in N7-N1 amino carbonyl and six G-G base pairs in N1-carbonyl symmetric geometry. The G-C and G-A base pairs form a cavity in the centre of the structure. On each side of the structure, two edgewise loops are stabilized by three G-G base pairs in N1-carbonyl symmetric geometry. In addition, two adenine residues are orientated inside the structure and are tightly packed with three G-G base pairs and two G-A base pairs.

We demonstrated that 31 nt sequence (VK1-A16-VK1), VK2, folds into a monomer, with structural features very similar to VK1. The only difference is an additional adenine residue A16 that formally connects the 5′ and 3′ ends of individual molecules of VK1. Since VK2 is located in the regulatory region of PLEKHG3 gene in the 14th chromosome, a likely candidate contributing to the risk of autism, the fold is potentially of huge biological relevance. In addition, VK1 and VK2 structures expand the repertoire of known DNA structural motifs and might also contribute to the design of programmable DNA folding.

## Methods

### Sample preparation

The isotopically unlabelled, residue-specific low-enrichment (6% ^15^N-labelled), residue-specific completely (100%) D8-labelled and VK1 oligonucleotides with uniformly ^15^N,^13^C-labelled guanine and cytosine residues as well as unlabelled VK2 oligonucleotides were synthesized on K&A Laborgeraete GbR DNA/RNA Synthesizer H-8. In all cases, standard phosphoramidite chemistry was used. Deprotection was done with the use of aqueous ammonia at 55 °C for 24 h. Residue-specific completely (100%) D8-labelled VK1 oligonucleotides were deprotected with ND_4_OD at room temperature for 72 h. Samples were purified and desalted with the use of Millipore Stirred Ultrafiltration Cell model 8010 and a Sephadex G25 column to give NMR samples with concentrations between 0.1 and 2.8 mM per strand. The samples were prepared in the presence of LiCl with varying concentrations from 10 to 100 mM as well as 100 mM concentrations of NaCl, KCl and NH_4_Cl. pH value was set to 6 with the use of LiOH and HCl. Extra care was taken to ensure that only the specified cations were present in NMR samples.

### NMR spectroscopic experiments

All NMR experiments were performed on Agilent-Varian NMR Systems 600 and 800 MHz spectrometers in the temperature range from 0 to 80 °C. The majority of spectra were recorded at 0 °C. 1D ^1^H, ^15^N-edited ^1^H experiments performed on 6% residue-specific ^15^N-labelled samples, NOESY with mixing times of 80, 100, 200 and 250 ms, ^15^N-edited, ^15^N,^13^C-filtered NOESY spectrum with mixing time of 100 ms performed on equimolar mixture of unlabelled and uniformly ^13^C, ^15^N-GC-labelled oligonucleotides, ROESY with mixing time 40 ms and TOCSY (total correlation spectroscopy) spectra with mixing time 20 ms were acquired in 90% H_2_O and 10% ^2^H_2_O. Double-quantum filtered-COSY, ^1^H–^31^P COSY and 1D ^31^P TOCSY spectra with mixing times of 40 and 80 ms were acquired in 100% ^2^H_2_O. Intranucleotide correlations between imino and H2 and H8 aromatic protons were acquired with the use of a jump-and-return HMBC (JRHMBC) experiment performed on an isotopically unlabelled sample. Diffusion experiments were performed using thirty different gradient strengths (0.49–29.06 G cm^−1^). NMR spectra were processed and analysed using VNMRJ (Agilent) and Sparky (UCSF) software.

### Stoichiometry determination

The NMR signal at *δ* ~12.6 p.p.m. characteristic for the imino proton of the G-C base pair started to appear and became more intense on lowering of the VK1 oligonucleotide concentration. This signal was not a part of the NMR fingerprint characteristic for the multimer, was well resolved and could be integrated. The plot of signal integrals of the multimer versus the corresponding single-strand values established the dimeric nature of the VK1 structure.

### CD spectroscopy

CD experiments were carried out on an Applied Photophysics Chirascan CD spectrometer over 180–330 nm and 200–320 nm wavelength ranges. All measurements were made in 0.1 and 0.25 cm path-length quartz cells. The oligonucleotide concentrations were between 0.1 and 1.0 mM.

### Ultraviolet spectroscopy

Ultraviolet spectra were acquired on a Varian Cary 100 Bio with 0.1, 0.2, 0.5 and 1.0 cm path-length cells. The extinction coefficients of VK1 and VK2 used were 155,200 and 322,200 M^−1^ cm^−1^, respectively.

### Restraints and structure calculations

Distance restraints used in structural calculations were obtained from the NOESY spectrum recorded at 80 ms mixing time in 90% H_2_O, 10% ^2^H_2_O. The H2′-H2″ cross-peaks of A8, A12, C6 and C14 residues could not be used as references because it was revealed that they experience a different NOE regime than cross-peaks in the rest of the spectrum. Cytosine H5-H6 cross-peak severely overlapped with the water signal and also could not be used as a reference. We had to rely on averaging the volumes of NOE correlations of H8 and H1′ protons observed between sequential residues. Because all the residues clearly exhibited an anti-orientation along glycosidic bonds, we could reference the averaged volumes to a value of 3.9 Å. With the help of this reference, we classified the remaining signals as strong (1.8−3.6 Å), medium (2.6−5.0 Å) and weak (3.5−6.5 Å). Torsion angle restraints along glycosidic bonds (torsion angle *χ*) for all residues were set between 170° and 310°, a value typical for residues in anti-orientation. The coupling constants gathered from the ^1^H–^31^P COSY spectrum enabled us to set the *ε* torsion angles of G1, G2 and G3 residues between 160° and 270° and the *β* torsion angles of G2, G3 and A4 residues between 70° and 100°. The initial starting structure consisted of two single strands, which were created using X3 DNA and the LEAP module of the AMBER 11 program^35^,^36^. Simulated annealing calculations were performed with AMBER 11 using the leap_parm99bsc0_chiOL4.in force field in the presence of explicit water^37^. SA calculations were initiated with random velocities. The SHAKE algorithm with a tolerance of 0.00005 Å for hydrogen atoms was used. The cut-off for nonbonded interactions was 8 Å. Ten lowest-energy structures calculated with SA were subjected to a maximum of 100,000 steps of steepest descent energy minimization. Figures were visualized and prepared with UCSF Chimera software^38^. The coordinates of the VK1 structure have been deposited in the Protein Data Bank with the accession code 2MJJ.

## Author contributions

V.K. performed the experiments. J.P. designed the study. V.K. and J.P. wrote the manuscript.

## Additional information

**How to cite this article:** Kocman, V. & Plavec, J. A tetrahelical DNA fold adopted by tandem repeats of alternating GGG and GCG tracts. *Nat. Commun.* 5:5831 doi: 10.1038/ncomms6831 (2014).

**Accession codes**: The coordinates of the VK1 have been deposited in the Protein Data Bank with the accession code 2MJJ.

## Supplementary Material

Supplementary Information Supplementary Figures 1-12

## Figures and Tables

**Table 1:** NMR restraints and structural statistics for the VK1 structure.

	**Non-exchangeable**	**Exchangeable**
*NOE-derived distance restraints*		
Intranucleotide NOEs	120	0
Sequential (*n*, *n*+1)	132	24
Long-range (*n*, >*n*+1)	16	32
Torsion angle restraints	70	
Hydrogen bond restraints	32	
		
*Structural statistics*		
NOE violations >0.3 Å	1	
		
*Deviations from idealized covalent geometry*		
Bonds (Å)	0.024±0.000	
Angles (deg)	2.641±0.041	
		
*Pairwise heavy-atom r.m.s.d. (Å)*		
Overall	1.121	
Only core of the structure; residues A4-G7 and A12-G15	1.081	
Only hydrophobic pocket; residues G7-A12	1.025	
Without G1-A4 and G15 residues	1.022	
Only G1-A4 residues	1.297	

r.m.s.d., root mean squared deviation.
