# Supplementary material for: A tetrahelical DNA fold adopted by tandem repeats of alternating GGG and GCG tracts
Source: Nat Commun. 2014 Dec 15;5:5831. doi: 10.1038/ncomms6831 (PMC4275592; doi:10.1038/ncomms6831)
Supplement: Supplementary Information — Supplementary Figures 1-12 [file ncomms6831-s1.pdf]

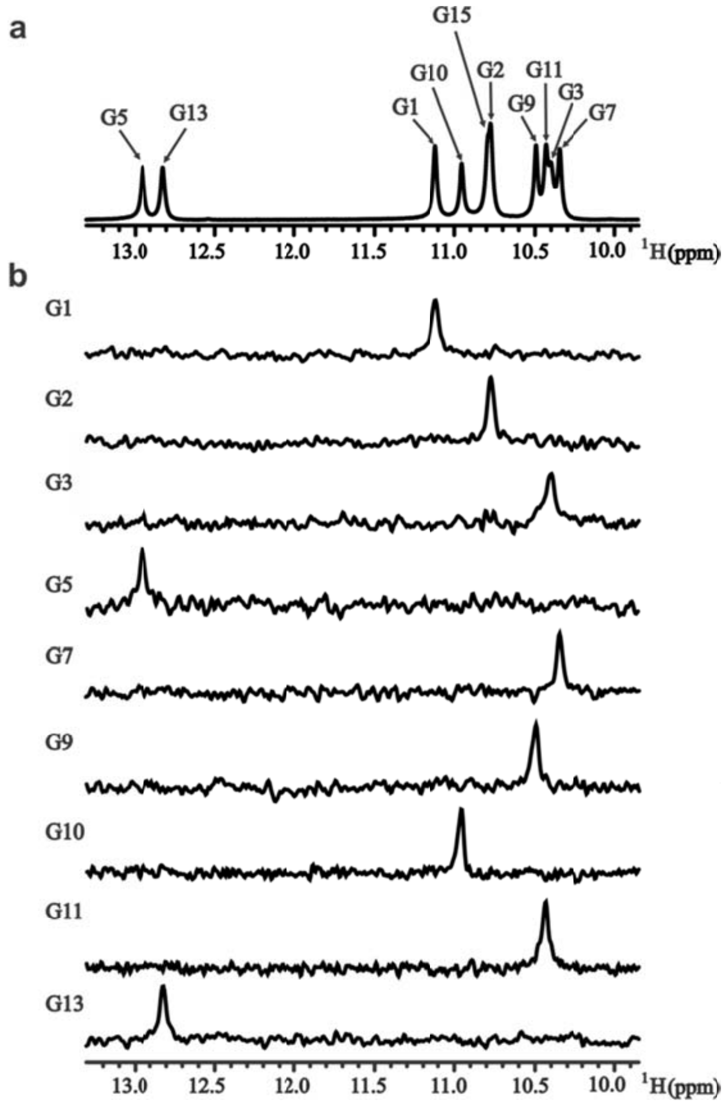

Supplementary Figure 1: Assignment of VK1 imino proton resonances. a, The imino region of ^{1}H NMR spectrum and assignment of guanine residues. **b**, 1D ^{15}N-edited HSQC spectra acquired on partially (6%) residue-specific ^{15}N-labeled oligonucleotides indicated next to individual spectrum. Spectra were recorded on 800 MHz (1D ^{1}H spectrum) and 600 MHz (1D ^{15}N-edited HSQC spectra) spectrometers at 0 °C, 100 mM LiCl and pH 6. Concentrations per strand were between 1 and 2.8 mM.

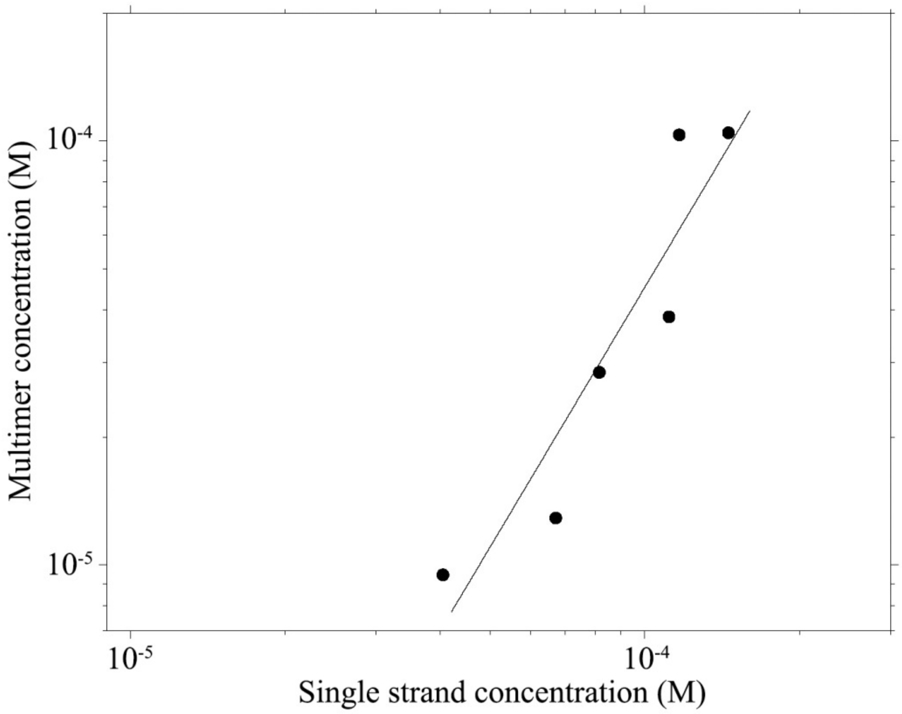

Supplementary Figure 2: Stoichiometry determination of VK1 oligonucleotide. Concentrations of folded multimer and single strand VK1 were determined by integration of well resolved imino NMR signals. The slope of the curve is 2.0 ± 0.5.

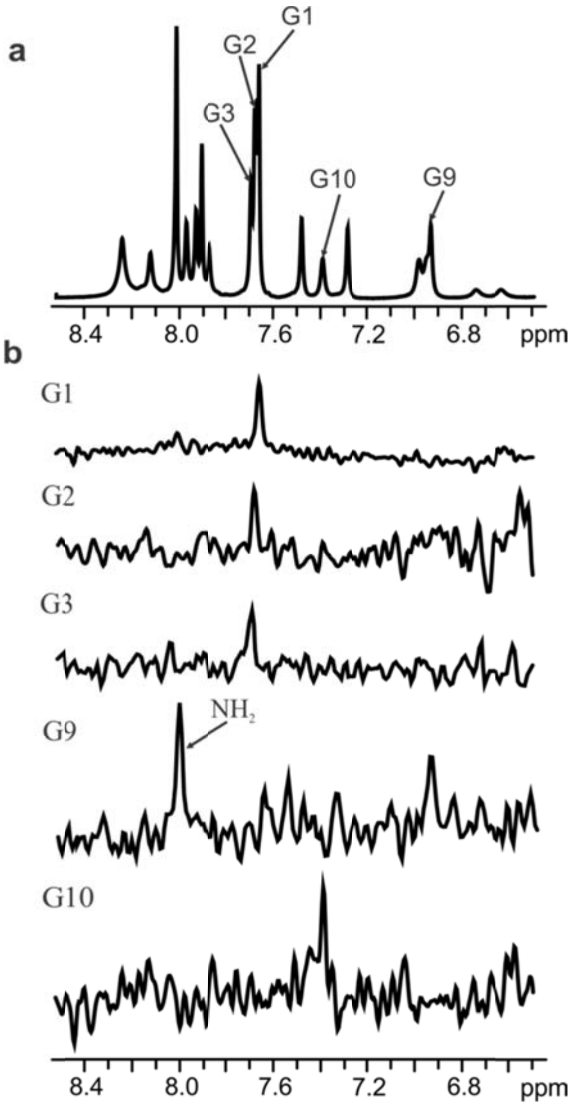

Supplementary Figure 3: Assignment of G1, G2, G3, G9 and G10 H8 proton resonances in VK1. a, Aromatic region of the 1D ^{1}H NMR spectrum and assignment of selected guanine residues. **b**, 1D ^{15}N-edited HMQC spectra acquired on partially (6%) residue-specific ^{15}N-labeled oligonucleotides indicated next to individual spectrum. Spectra were recorded on 800 MHz spectrometer at 0 °C, 100 mM LiCl and pH 6. Concentrations per strand were between 1.0 and 2.8 mM.

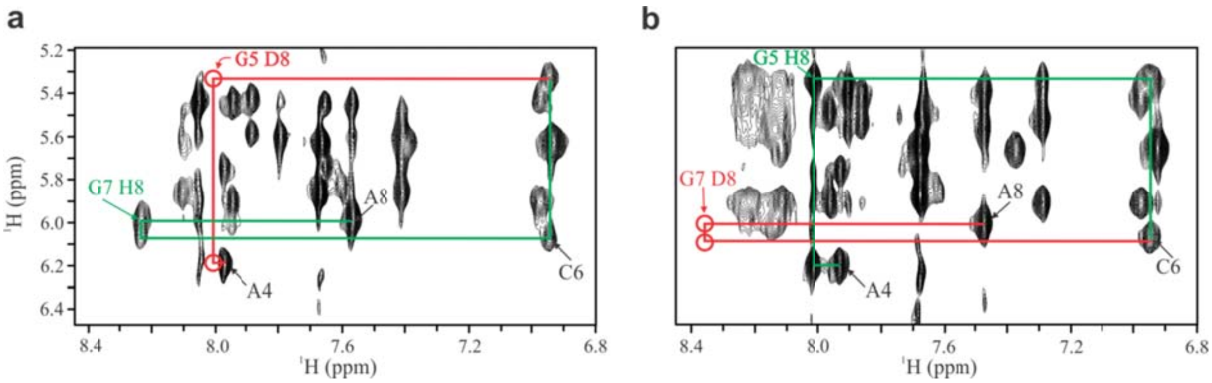

Supplementary Figure 4: NOESY spectra of C8-deuterium labeled guanine residues of VK1 at positions G5 (a) and G7 (b). The red lines show NOE connectivities that are absent due to the introduction of deuterium in residue-specific labeled VK1. The red circles indicate the place where the missing cross-peaks should be located. The green lines show sequential NOE connectivities of G7 in G5-deuterium labeled VK1 (**a**), and of G5 residue in G7-deuterium labeled VK1 (**b**). Spectra were recorded on an 600 MHz spectrometer at 0 °C, 20 mM LiCl and pH 5. Concentration per strand was 1.1 (**a**) and 1.4 mM (**b**).

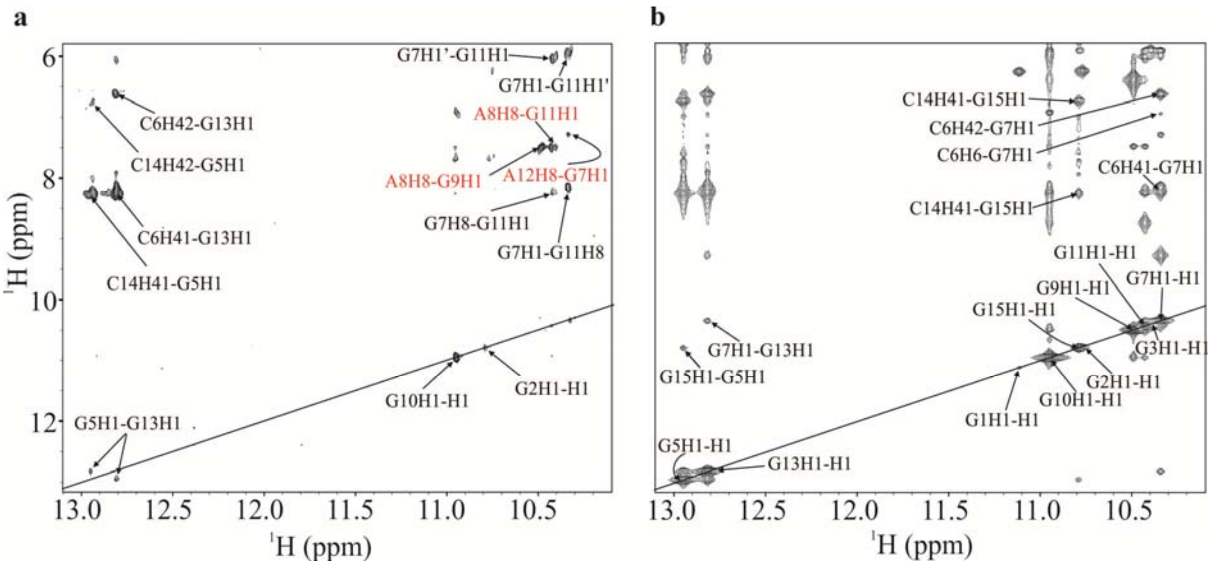

Supplementary Figure 5: The imino-imino regions of ^{15}N-edited, ^{15}N,^{13}C-filtered NOESY spectrum of VK1 that contains an equimolar mixture of unlabeled and uniformly ^{13}C, ^{15}N-GC-labeled oligonucleotides (a) and ^{1}H-^{1}H NOESY spectrum of VK1 (b). ^{15}N-edited, ^{15}N,^{13}C-filtered NOESY spectrum (**a**) was recorded at 0.9 mM concentration per strand, 0 °C, pH 6 and mixing time of 100 ms. ^{1}H-^{1}H NOESY spectrum (**b**) was recorded at 2.8 mM concentration per strand, pH 6.0, 0 °C and mixing time of 250 ms. The assigments are indicated. The red assignments highlight the NOE contacts that involve the adenine residues. For clarity, only signals that are not present in spectrum (**a**) are marked in spectrum (**b**).

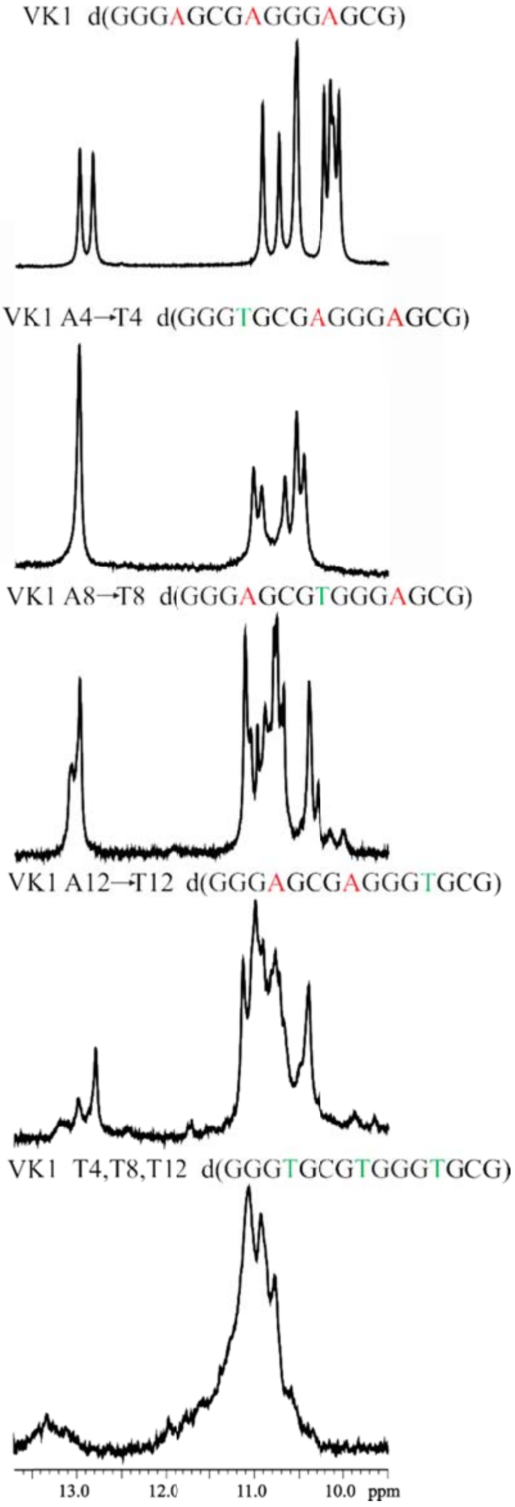

Supplementary Figure 6: Mutants of VK1 with A4 to T4, A8 to T8 and A12 to T12 substitutions. For easier identification A are labeled in red and T in green**.** All spectra were recorded at 0 °C, pH 6 and 1 mM concentration per strand.

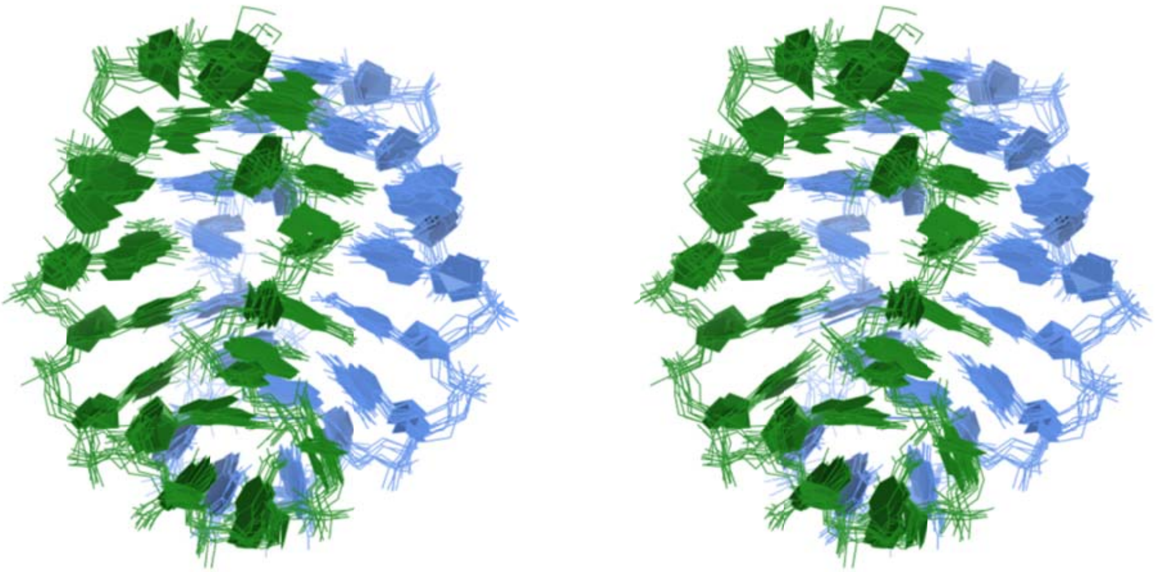

Supplementary Figure 7: Stereoscopic view of the 10 refined superimposed structures of VK1. Two sets of monomeric units are colored in green and blue.

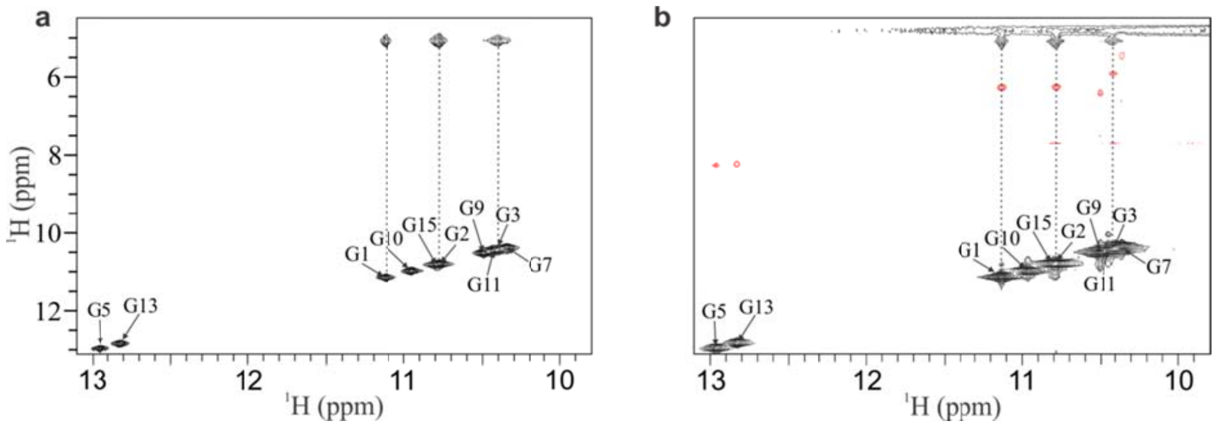

Supplementary Figure 8: Imino-imino region of TOCSY (a) and ROESY (b) spectra of VK1. The assignment of imino protons is shown. The vertical dashed lines connect autocorrelation imino proton signals with cross-peaks with bulk water. Spectra were recorded on an 600 MHz NMR spectrometer at 0 °C, 100 mM LiCl and pH 6. Concentration per strand was 2.8 mM.

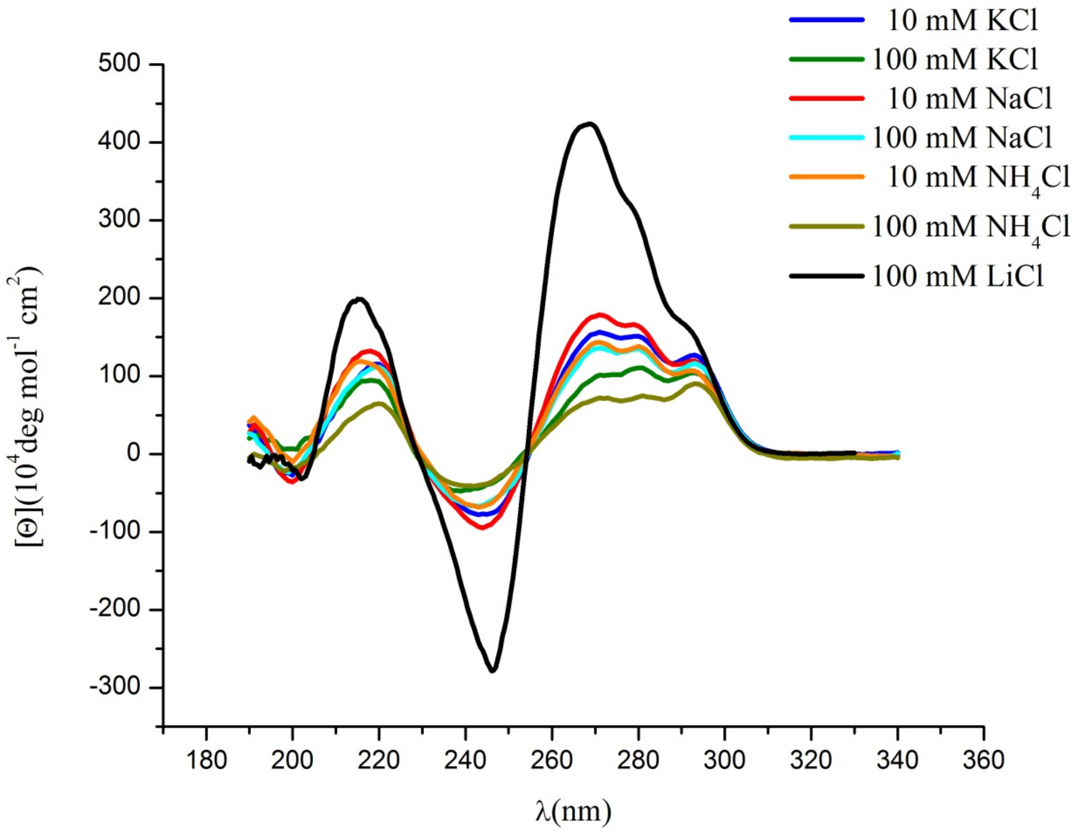

Supplementary Figure 9: CD spectra of VK1 in the presence of different salts and their concentrations. CD spectra were recorded at 0.8 mM concentration per strand in a 0.01 cm path-length cell at 1 °C, pH 6 and different salt concentrations indicated by different colors.

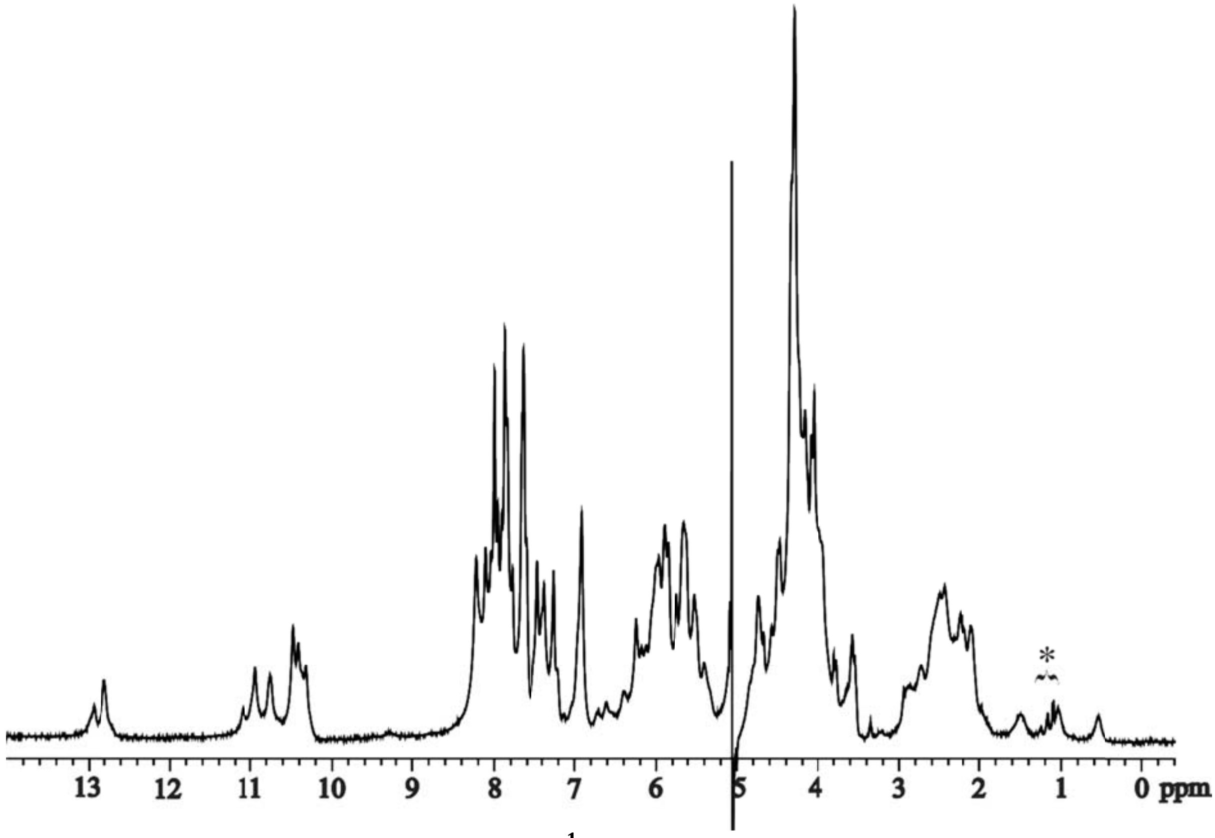

Supplementary Figure 10: The complete ^{1}H NMR spectrum of VK2. The spectrum was recorded at 1.1 mM oligonucleotide concentration per strand, 100 mM LiCl, pH 6 and 0 °C on an 800 MHz spectrometer. Signals of unknown impurities are labeled with *.

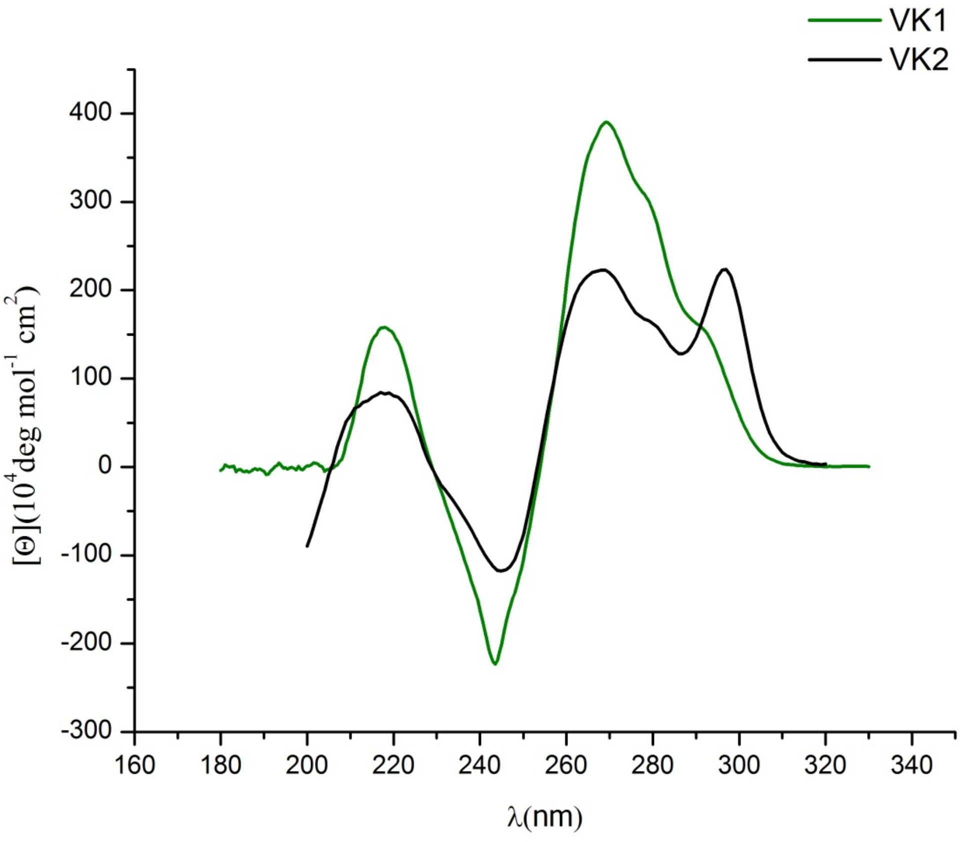

Supplementary Figure 11: CD spectra of VK1 and VK2. Both spectra were recorded at 1 mM concentration per strand, 0 °C, 100 mM LiCl and pH 6.

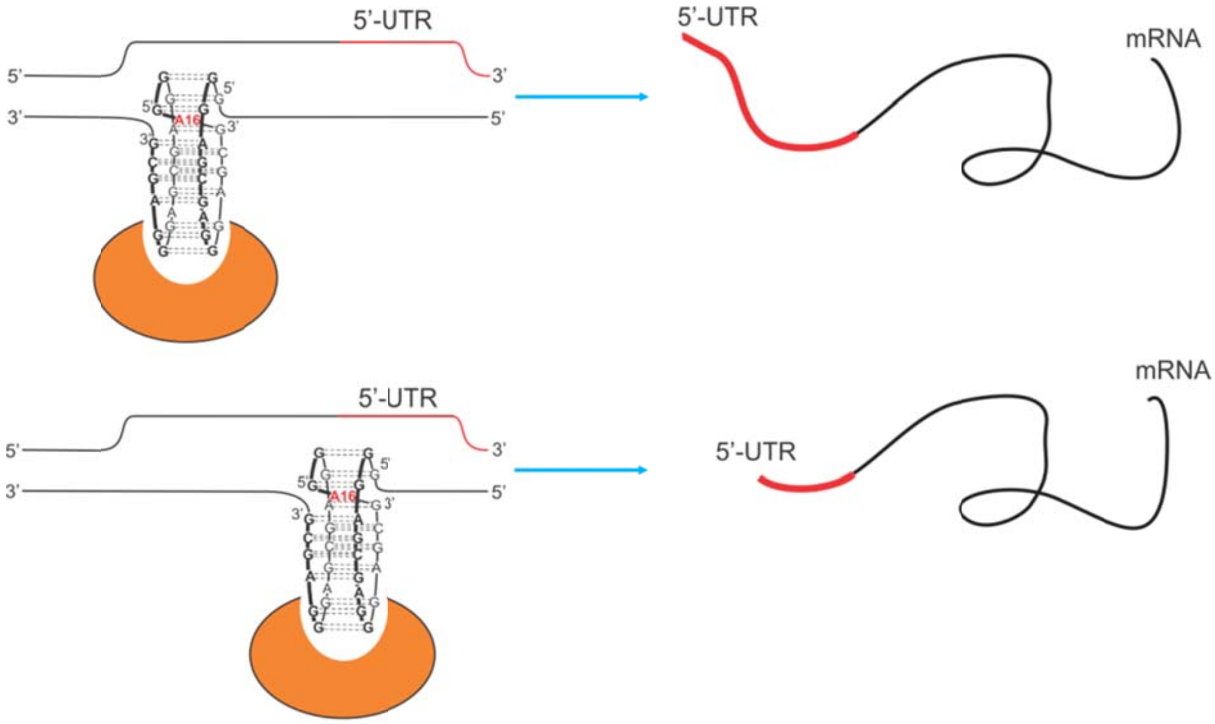

Supplementary Figure 12: A model showing VK2 folds at different positions in the regulatory region of the PLEKHG3 gene which have an impact on the length of the 5'-UTR region of the transcribed mRNA. Proteins of the transcription machinery that interact with promoter region are colored in orange.
